# The road to micronutrient biofortification of rice: progress and prospects

**DOI:** 10.3389/fpls.2013.00015

**Published:** 2013-02-08

**Authors:** Khurram Bashir, Ryuichi Takahashi, Hiromi Nakanishi, Naoko K. Nishizawa

**Affiliations:** ^1^Laboratory of Plant Biotechnology, Department of Global Agricultural Sciences, Graduate School of Agricultural and Life Sciences, The University of TokyoTokyo, Japan; ^2^Research Institute for Bioresources and Biotechnology, Ishikawa Prefectural UniversityIshikawa, Japan

**Keywords:** biofortification, biosafety, iron, micronutrient transport, *Oryza sativa* L., zinc

## Abstract

Biofortification (increasing the contents of vitamins and minerals through plant breeding or biotechnology) of food crops with micronutrient elements has the potential to combat widespread micronutrient deficiencies in humans. Rice (*Oryza sativa *L.) feeds more than half of the world’s population and is used as a staple food in many parts of Asia. As in other plants, micronutrient transport in rice is controlled at several stages, including uptake from soil, transport from root to shoot, careful control of subcellular micronutrient transport, and finally, and most importantly, transport to seeds. To enhance micronutrient accumulation in rice seeds, we need to understand and carefully regulate all of these processes. During the last decade, numerous attempts such as increasing the contents/expression of genes encoding metal chelators (mostly phytosiderophores) and metal transporters; Fe storage protein ferritin and phytase were successfully undertaken to significantly increase the micronutrient content of rice. However, despite the rapid progress in biofortification of rice, the commercialization of biofortified crops has not yet been achieved. Here, we briefly review the progress in biofortification of rice with micronutrient elements (Fe, Zn, and Mn) and discuss future prospects to mitigate widespread micronutrient deficiencies in humans.

## INTRODUCTION

Micronutrients are not only essential for plant growth and development but are also integral to human and animal health. In the last two decades, the concept of hidden hunger (deficiency of certain vitamins and micronutrient nutrients despite eating enough calories) has been well established (Nilson and Piza, 1998). As a result, the importance of micronutrient nutrition is increasing at a great pace. The micronutrients iron (Fe), zinc (Zn), and manganese (Mn) are of particular interest, given that all three are essential micronutrients for all higher organisms and we will focus on these three micronutrients in this review. Fe serves as an important cofactor for various enzymes performing basic functions in humans. Fe deficiency results in anemia and is also reported to have pathological consequences (Stoltzfus, 2003; Hentze et al., 2004). Zn deficiency causes growth retardation, hypogonadism, immune dysfunction, and cognitive impairment (Prasad, 2009). Fe and Zn deficiencies are among the most prevalent micronutrient deficiencies in humans, affecting two billion people and causing more than 0.8 million deaths annually (World Health Organization, 2003). Mn deficiency is also a serious problem and can lead to asthma and severe birth defects, however, it is relatively less prevalent compared to Fe and Zn deficiency. The combined effects of these micronutrient deficiencies pose a significant threat to human health.

Biofortification (increasing the contents of vitamins and micronutrients through plant breeding or biotechnology) of food crops with vitamins and micronutrients is not a new concept and was suggested more than a decade ago as a way to significantly ameliorate deficiencies (Ye et al., 2000; Guerinot, 2001; Clemens et al., 2002b). Biofortification is an effective and cheaper alternate to traditional ways of combating micronutrient deficiencies, i.e., healthy food (which is often expensive), micronutrient supplementation, and food fortification. These conventional methods are difficult to afford for a large proportion of the world’s population, especially those with limited resources and low incomes (Haas et al., 2005). Increasing the micronutrient content of grain by biofortification offers great potential to combat micronutrient deficiency and dramatically impact human health. Another definition of the problem is nutritional genomics which is described as manipulating plant micronutrients to improve human health by the interface of plant biochemistry, genomics, and human nutrition (DellaPenna, 1999).

Fe, Zn, and Mn are also essential in plants. Fe participates in various cellular events such as respiration, chlorophyll biosynthesis, and photosynthetic electron transport. Low chlorophyll content (chlorosis) of young leaves is the most obvious visible symptom of Fe deficiency (Marschner, 1995). Fe deficiency also seems to triggers oxidative stress (Tewari et al., 2005; Bashir et al., 2007). Fe is essential for the function of chloroplast and mitochondria. Fe is transported to mitochondria through mitochondrial Fe transporter (MIT; Bashir et al., 2011a,c). Additional Fe may be diverted to vacuole (Kim et al., 2006; Zhang et al., 2012). In rice the vacuolar Fe transporter 1 (OsVIT1) and OsVIT2 are suggested to play an important role in subcellular Fe transporter (Zhang et al., 2012).

Zn plays diverse roles in different cellular processes (Ishimaru et al., 2011b). Protein, nucleic acid, carbohydrate, and lipid metabolism depend to a great extent on Zn (Rhodes and Klug, 1993; Vallee and Falchuk, 1993). Zn uptake must be tightly regulated to ensure that the correct amount of Zn is present at all times (Ishimaru et al., 2011b). In plants, Zn deficiency results in the accumulation of starch and inactive RNases, suggesting that RNA degradation could be regulated by the availability of Zn in the cell (Suzuki et al., 2012). Mn serves as a cofactor or activator of enzymes with different functional groups and diverse activities. For example, oxalate oxidase, Mn superoxide dismutase, RNA polymerase, malic enzyme, isocitrate dehydrogenase, and phosphoenolpyruvate carboxykinase use Mn as a cofactor (Bowler et al., 1991; Marschner, 1995; Requena and Bornemann, 1999; Alscher et al., 2002). Mn is also important in photosynthetic oxygen evolution in chloroplasts (Britt, 1996; Clemens et al., 2002a; Rutherford and Boussac, 2004). Mn-deficient plants are more susceptible to low-temperature stress and pathogen infection, leading to significant decreases in crop yield (Marschner, 1995; Hebbern et al., 2005). The transport of Mn is believed to share an entry route with Fe and cadmium (Cd). Fe, Zn, and Mn transport overlaps in plant biological systems, and Fe, Zn, and Mn deficiencies are particularly severe when plants are grown in alkaline soils. As alkaline soils account for approximately 30% of the world’s cultivated soils (Chen and Barak, 1982), the development of plants able to grow in these soils may greatly benefit agriculture. Furthermore, plants that accumulate more micronutrients (e.g., Fe, Zn, and Mn) would contribute significantly to combating micronutrient deficiencies in humans.

## SEED MICRONUTRIENT LOCALIZATION AND BIOFORTIFICATION OF RICE

In rice seeds Fe localizes to dorsal vascular bundle, aleurone layer, and endosperm as well as it localizes to the scutellum and vascular bundle of the scutellum of embryo. During germination localization of Fe changes significantly, particularly in embryo and 36 h after sowing, Fe localizes to the scutellum, coleoptile, and epithelium, as well as to the leaf primordium and radicle (Takahashi et al., 2009). Unlike Fe, Zn is unevenly distributed to all of parts of the seed, with a significantly high value for the aleurone layer and embryo (Takahashi et al., 2009). During germination, Zn flow is quite dynamic compared to Fe and Mn. Mn is observed both in the endosperm and embryo, and during germination accumulation of Mn increases in the scutellum at the cost of Mn accumulation in coleoptile. Besides micronutrients, rice seed also contain nicotianamine (NA) and 2′-deoxymugineic acid (DMA; Masuda et al., 2009; Usuda et al., 2009), which are suggested to chelate and mobilize micronutrients during germination (Takahashi et al., 2009).

Significant variation for seed Fe concentration has been reported in rice (Gregorio et al., 2000). Fe is abundant in mineral soils and the major problem with its acquisition is solubility, thus application of soil Fe as fertilizer is not an effective strategy for increasing seed Fe. The application of Zn as fertilizer is effective in promoting plant growth and also in the fortification of crops with Zn. Variation in different rice genotypes in terms of Zn use efficiency and grain Zn concentration has been reported (Neue et al., 1998; Yang et al., 1998; Graham et al., 1999; Wissuwa et al., 2006, 2008). For controlling grain Zn concentrations, the native soil Zn status is the dominant factor, followed by genotype and fertilizer, while for Fe pH of the soil and the concentration of carbonate as well as field conditions like submergence or dry filed are more important. Rice grain Zn concentrations may be as low as 15.9 and as high as 58.4 mg kg^-^^1^ depending upon the genotype (Graham et al., 1999), and within a single variety, the grain Zn concentration may vary from 8 to 47 mg kg^-^^1^ depending on soil Zn status (Wissuwa et al., 2006, 2008). Different labs have demonstrated the potential to increase the Zn concentration of rice grains through soil–plant interactions, traditional breeding, and marker-assisted breeding (reviewed by Impa and Johnson-Beebout, 2012); however, traditional breeding has thus far been an unsuccessful approach for increasing the seed Fe concentration.

## BIOFORTIFICATION THROUGH BIOTECHNOLOGY

Upon storage, especially in tropical environments, rice seeds rapidly deteriorate. As a conventional practice for maintaining the quality and improving the shelf-life of rice grains, the embryo, pericarp, testae, and aleurone layers are removed during milling, leaving only the endosperm as the edible part (Matsuo and Hoshikawa, 1993). Thus, biofortification would only be effective when metal concentrations are increased in the rice endosperm. Biotechnology techniques such as plant transformation offer great opportunities for increasing the amounts of trace metals in the endosperm. **Table 1** reviews the types of genes that have been used for plant transformation in attempts to increase the metal content of rice endosperm. The genes used for biofortification of rice are mainly those encoding metal chelators (mostly phytosiderophores) and metal transporters; genes encoding the Fe storage protein ferritin and phytase have also been used.

**Table 1:** Summary of different approached undertaken for mineral biofortification of rice.

Gene	Promoter	Cultivar	Fold increase in Fe/Zn	Reference
*AtNAS1+, Pvferritin+, Afphytase*	CaMV 35S, Glb-1, Glb-1	*Japonica* cv. Taipei 309	6.3/1.6^1^	Wirth et al. (2009)
*HvNAS1*	Actin	*Japonica* cv. Tsukinohikari**	3.4/2.3^2^	Masuda et al. (2009)
*HvNAS1, HVNAS1+HvNAAT, IDS3*	Genomic fragments	*Japonica* cv. Tsukinohikari**	1.0/1.0^2^, 1.1/1.1, 1.4/1.3	Masuda et al. (2008)
*OsNAS1, OsNAS2, OsNAS3*	CaMV 35S	*Japonica* cv. Nipponbare	2.2/1.4, 4.2/2.2, 2.2/1.4	Johnson et al. (2011)
*OsNAS2*	Activation tagging	*Japonica* cv. Dongjin	3/2.7	Lee et al. (2011, 2012b)
*OsNAS3*	Activation tagging	*Japonica* cv. Dongjin	/2.2	Lee et al. (2009b)
*OsNAS1*	GluB1	*Japonica* cv. Xiushui 110**	1.0/1.3	Zheng et al. (2010)
*SoyferH-1*	Glu-B1	*Japonica* cv. Kitaake	3.0	Goto et al. (1999)
*PvFerritin+ rgMT*	Glb-1	*Japonica* cv. Taipei 309	2.0^3^	Lucca et al. (2001)
*SoyFer*	Glu-B1	*Indica* cv. IR68144	3.7/1.4	Vasconcelos et al. (2003)
*SoyFer*	Glu-B1; Glb-1	*Japonica* cv. Kitaake	3.0/1.1	Qu et al. (2005)
*OsFer2*	*OsGluA*2	Basmati rice (*Indica* cv. Pusa-Sugandh II)	2.1/1.4	Paul et al. (2012)
*TOM1*	CaMV 35S	*Japonica* cv. Tsukinohikari	1.2/1.6	Nozoye et al. (2011)
*OsYSL2*	OsSUT1	*Japonica* cv. Tsukinohikari	4.4	Ishimaru et al. (2010)
*OsIRT1*	Ubi	*Japonica* cv. Dongjin	1.1/1.1^3^	Lee and An (2009)
*OsYSL2+, SoyFerH2+, HvNAS1*	OsSUT1, Glb-1, Glb-1. Glu-B1, Act	*Japonica* cv. Tsukinohikari	6 or 4^4^/1.6	Masuda et al. (2012)

1 Mn concentration also increased by two fold.

2 Plants were tested in isolated filed.

3 Concentration in brown rice.

4 Six times in T2 seeds while 4.2 times in T3 seeds.

### BIOFORTIFICATION THROUGH INCREASING THE AMOUNT OF METAL CHELATORS

Graminaceous plants, which include rice, have sophisticated mechanisms for acquiring micronutrients from soil and transporting them from roots to shoots and grains by secreting small molecules called mugineic acid family phytosiderophores (MAs). MAs have the potential to solubilize Fe, Zn, Cu, and Mn (Treeby et al., 1989). The synthesis of MAs has been studied at the molecular level and has been extensively reviewed (Bashir et al., 2006, 2010, 2012; Bashir and Nishizawa, 2006; Nagasaka et al., 2009; Ishimaru et al., 2011b; Kobayashi and Nishizawa, 2012; Suzuki et al., 2012); thus, it will be discussed very briefly here. MAs are synthesized from L-Met (Mori and Nishizawa, 1987) via a conserved pathway comprising the trimerization of *S*-adenosyl methionine to NA by NA synthase (NAS; e.g., OsNAS1-3), conversion of NA to a keto-intermediate by NA aminotransferase (NAAT; e.g., OsNAAT1), and transformation of the keto-intermediate to 2′-DMA by DMA synthase (DMAS; e.g., OsDMAS1). NA is also a metal chelator and is found in all plants. MAs are released into rhizosphere through transporter of mugineic acid 1 (TOM1), a member of major facilitator superfamily antiporter (Nozoye et al., 2011) and the metal-MA complex is taken up by transporters belonging to the yellow stripe-like (YSL) family (Curie et al., 2001; Inoue et al., 2009). Exporter of NA 1 (ENA1) is NA transporter suggested to be localized to vacuole (Nozoye et al., 2011). ENA1 is similar to the *Arabidopsis* *thaliana* zinc-induced facilitator 1 (AtZIF1) and AtZIFL2. AtZIF1 also localizes to vacuolar membrane and is suggested to be involved in Zn detoxification through sequestration into the vacuole (Haydon and Cobbett, 2007). Increasing the concentrations of NA and DMA in rice plants has been shown to effectively increase the metal concentration in rice grains. The Fe and Zn concentrations in rice grains were significantly increased by the overexpression of *HvNAS1* (Masuda et al., 2012, 2008) or *OsNAS1-3* (Johnson et al., 2011), the simultaneous overexpression of *AtNAS1* and *Pvferritin* (Wirth et al., 2009), and the activation of *OsNAS2* and *OsNAS3* (Lee et al., 2009b, 2011, 2012b). The overexpression of *TOM1* slightly increased seed Fe, Zn, and Cu concentrations (Nozoye et al., 2011), and the overexpression of barley iron deficiency-specific clone 3 (*IDS3*), whose product converts DMA to MA and 3-epihydroxy-2′-deoxymugineic acid (epiHDMA) to 3-epihydroxymugineic acid (epiHMA) (Nakanishi et al., 2000), increased Fe accumulation in rice grains (Masuda et al., 2008). These results suggest that an increase in NA and/or DMA/MA synthesis could increase Fe and Zn translocation to rice grains and that the increase in Fe is positively correlated with the accumulation of NA or DMA (Johnson et al., 2011; Masuda et al., 2012).

### EXPLOITING METAL TRANSPORTERS FOR BIOFORTIFICATION

Metal transporters belonging to different families have been reported to play significant roles in metal uptake in rice; these have been discussed extensively (Koike et al., 2004; Ishimaru et al., 2007, 2011a,b,c, 2012a,b; Lee and An, 2009; Lee et al., 2009a, 2010a,b, 2012a,c; Bashir et al., 2010, 2011a,b,c, 2012; Kakei et al., 2012; Suzuki et al., 2012). In short, iron-regulated transporter-like protein 1 (OsIRT1; Fe and Cd), OsIRT2 (Fe and Cd), natural resistance-associated macrophage protein 1 (OsNRAMP1; Fe and Cd), and OsNRAMP5 (Mn, Fe, and Cd) transport metals (Bughio et al., 2002; Ishimaru et al., 2006, 2012a,b; Nakanishi et al., 2006; Takahashi et al., 2011; Sasaki et al., 2012), while OsYSL2 (Mn–NA or Fe–NA), OsYSL15, OsYSL16, and OsYSL18 [Fe(III)–DMA] transport NA- or DMA-bound metals (Koike et al., 2004; Aoyama et al., 2009; Inoue et al., 2009; Lee et al., 2009a, 2012c; Ishimaru et al., 2010; Kakei et al., 2012). Moreover, the rice phenolics efflux transporters, phenolics efflux zero 1 and 2 (PEZ1 and PEZ2), secrete phenolics in to apoplasm (in roots and in xylem) to solubilize apoplasmic Fe for transport (Bashir et al., 2011b; Ishimaru et al., 2011a,c). All these transporters localize to plasma membrane and transport apoplasmic micronutrients into cytoplasm. Although OsIRT1, OsIRT2, OsYSL15, and OsNRAMP5 are mainly involved in micronutrient uptake from soil, these transporters are also suggested to play a role in micronutrient translocation to seeds. On the other hand, OsYSL2, OsYSL16, and OsYSL18 are suggested to be involved in xylem to phloem transport (phloem loading) in rice while TOM1 may also play a role in xylem loading (Kobayashi and Nishizawa, 2012).

Increases in the uptake of Fe from the soil and its translocation through the plant are reported to increase the Fe content of rice endosperm. Rice lines that overexpress *OsIRT1* accumulated more Fe and Zn in the seeds (Lee and An, 2009), and similar results were observed for lines overexpressing *OsYSL15* (Lee et al., 2009a). The overexpression of *HvYS1* in rice enhanced the tolerance to alkaline soil, but did not increase the Fe concentration in the grain (Gómez-Galera et al., 2012). *OsYSL2* overexpression resulted in decreased root-to-shoot translocation of metals, whereas *OsYSL2* overexpression driven by a sucrose transporter promoter (*OsSUT1*) which specifically expresses in phloem, significantly increased Fe and Mn concentrations in rice seeds (Ishimaru et al., 2010). Thus, especially for metal transporters, control of the spatial and temporal expression of genes may significantly increase Fe flow to rice grains.

Additional transporters also appear to be good candidates for metal biofortification. Overexpression of rice plasma membrane zinc-regulated transporter (ZRT) IRT like protein 4 (OsZIP4), *OsZIP5*, and *OsZIP8* resulted in decreased root-to-shoot translocation of Zn and reduced seed Zn concentrations (Ishimaru et al., 2007; Lee et al., 2010a,b). The inclusion of tissue-specific promoters such as the *OsSUT1* promoter, which was used in the case of *OsYSL2* (Ishimaru et al., 2010) may increase the Zn concentration in rice grains. Rice plants overexpressing rice *heavy metal ATPase 2* (*OsHMA2*) under the control of *OsSUT1* were reported to accumulate slightly more Zn in seeds (Takahashi et al., 2012).

### OTHER APPROACHES FOR MICRONUTRIENT BIOFORTIFICATION

Ferritin, a globular protein found in prokaryotes and eukaryotes, has the ability to store and keep Fe in a soluble and non-toxic form (Harrison and Arosio, 1996). In plants ferritin is mainly localized to plastids (Briat et al., 2010). The overexpression of ferritin was the first attempt at increasing the metal content of rice grains. Ferritin-overexpressing plants accumulated Fe in seed endosperm at a level threefold that in wild-type plants (Goto et al., 1999; Lucca et al., 2001; Vasconcelos et al., 2003; Qu et al., 2005). Recent approaches employed to increase the solubility of Fe include the development of rice plants expressing *Aspergillus fumigatus* thermotolerant phytase and a cysteine-rich metallothionein-like protein (Lucca et al., 2001). Phytases are abundant in grains and oil seeds and play an important role in the degradation of phosphates containing organic molecules (such as phytate; Li et al., 2010), thus increasing the solubility of Fe. Metallothioneins are low molecular weight proteins rich in cysteine and are suggested to play a role in regulation of metals by binding to these metals (Grennan, 2011).

In addition, phytase overexpression with the aim of degrading phytic acid was attempted in order to increase micronutrient bioavailability in rice (Wirth et al., 2009). Basmati rice (Pusa-sugandh II) plants overexpressing rice ferritin (*OsFer2*) under the control of the endosperm-specific *GlutelinA2* (*OsGluA*2) promoter were established (Paul et al., 2012). The expression of ferritin in these T_3_ transgenic plants was 7.8-fold than that in wild-type plants, and the transgenic plants accumulated Fe and Zn at levels 2.1- and 1.4-fold, respectively, compared to wild-type plants (Paul et al., 2012).

Furthermore, the simultaneous overexpression of ferritin under the control of the endosperm-specific promoters *globulin b1* (*OsGlb1*) and *glutelin B1 *(*OsGluB1*), *NAS* under the control of the *OsActin1* promoter, and *OsYSL2* under the control of *OsGlb1* promoter and the *OsSUT1* transporter promoter have been demonstrated to significantly increase the Fe, Zn, Mn, and Cu concentrations in T_3_ polished seeds of field grown rice (Masuda et al., 2012).

## BOTTLENECK OF BIOFORTIFICATION

The application of Fe transporters for micronutrient biofortification of rice is problematic because many transporters, including OsIRT1, OsNRAMP1, and OsNRAMP5, also transport other metals such as Cd. Cd is a toxic metal in humans (World Health Organization, 2003) and affects cellular metabolism (Singh and McLaughlin, 1999). Thus, the use of these transporters for biofortification may also increase the grain Cd concentration in rice grown in soils contaminated with Cd. The knockout of *PEZ1* and *PEZ2* increased the seed Cd content, and *PEZ1* overexpression significantly reduced plant growth, probably due to Fe toxicity (Bashir et al., 2011b; Ishimaru et al., 2011a,c). It is possible to use mutated versions of these proteins that have narrow substrate specificity and do not transport Cd. It has been demonstrated that replacing specific amino acids in *Arabidopsis* IRT1 changed its substrate specificity (Rogers et al., 2000). Moreover, Podar et al. (2012) recently revealed the key components controlling metal selectivity, thus offering a strategy for the modification of transporters for effective biofortification.

The physiology and seed morphology of a particular crop plant should be carefully considered before utilizing any gene for biofortification. A gene that is effectively used in one crop may not be suitable for use in other crops, and vise versa. This is especially true in the case of *OsVIT1* and *OsVIT2*. Although *osvit1-1* and *osvit2-1* mutants accumulate significantly higher levels of Fe and Zn in brown rice, most of the Fe localizes to the embryo and aleurone layer (Zhang et al., 2012), which are removed during the processing and polishing of rice grains. Moreover, although OsVIT1 and OsVIT2 do not transport Cd, the grain Cd concentration increased by 60% in rice seeds of *osvit1-1* and *osvit2-1* mutants compared with the concentration in wild-type plants when grown in Cd-contaminated soils. Thus, these genes do not offer an advantage for the biofortification of rice. In rice the role of other subcellular metal transporters, such as MIT, in regulating seed metal concentration is not clear. It may be better to consider regulating the expression of these transporters to exploit their full potential for increasing the micronutrient concentration of grains.

Careful examination and understanding of the expression patterns of the genes involved in micronutrient transport throughout plant development are important before selecting a gene for use in a biofortification program. This information would help to identify candidate genes that could significantly enhance the micronutrient content in rice grains as well as to develop rice plants that are tolerant to low micronutrient availability under diverse environmental conditions. Based on the expression patterns of genes in rice, it appears that OsYSL16 and its promoter may be good candidate for increasing the micronutrient concentration in rice grains.

## FUTURE PROSPECTS

The ultimate objective of developing biofortified crops is to utilize these crops in the field; however, no serious efforts have been reported for the commercialization of biofortified rice. For example, although sufficient scientific research and development have led to the establishment of stable transgenic lines of golden rice, a rice biofortified with β-carotene, the commercialization of golden rice has suffered from serious delays (Potrykus, 2012). Thus far,transgenic rice has not been commercialized in any country. It is possible that after the initial commercialization of golden rice, commercialization of micronutrient-biofortified rice will proceed at a faster rate, depending upon its acceptance by farmers and its performance in the field. Furthermore, the use of genes from rice or other plants to produce biofortified rice such as golden rice may meet with consumer approval more easily than the use of *Bacillus thuringiensis* (*Bt*) genes. It is unfortunate that exploitation of non-transgenic mutants, such as Tos17 mutants or mutants generated through chemical or radiation treatment, has not been examined for the micronutrient biofortification of rice.

Rice feeds more than half of the world’s population and is a staple food in most parts of Asia. When considering the commercialization of biofortified rice, it should be remembered that most rice is consumed in Asia and that many of the potential beneficiaries belong to poor countries and poor families. Thus, regulatory laws that prevent the mixing of genetically modified rice with traditional rice may pose practical impediments. Many countries export rice to other countries, and it may be difficult to enforce regulatory laws in the field, which could lead to problems for rice exporters. In addition, the suitability of sites for the cultivation of biofortified transgenic rice must be evaluated with regard to maintaining biodiversity and fully exploiting the potential of transgenic rice. Biosafety issues concerning different traits of transgenic rice have been discussed extensively (Husnain et al., 2003, 2004; Bashir et al., 2004a,b, 2005; Riaz et al., 2006). One important issue is the development of marker-free lines. Recently, rice lines defective in the MIT (Bashir et al., 2011a,c) were complemented using a mutated rice acetolactate synthase gene as a selectable marker (Ogawa et al., 2008). Markers that are originally cloned from rice may be easily accepted by farmers. In addition, lines harboring the *HvNAS1* gene and accumulating higher amounts of NA have been developed (Usuda et al., 2009), and the Cre/*loxP* DNA excision (CLX) system was successfully used to remove the hygromycin resistant (HPT) marker gene in these lines. As a result, the final transformants were marker-free and contained the *HvNAS1* gene alone (Usuda et al., 2009). Moreover, these transgenic lines were crossed with a cleistogamous mutant to prevent gene transfer through cross-pollination. Although the rate of cross-pollination in transgenic rice is very low (Bashir et al., 2004a; Mahmood-ur-Rahman et al., 2007), it is still wise to utilize cleistogamous mutants to further reduce this possibility. The development of marker-free and cleistogamous rice would help to minimize public concern with regard to the use of transgenic rice in biofortification programs. The number of transgenes in a line may also create public objections. Transgenic lines containing one gene (e.g., *OsNAS2*; Johnson et al., 2011) appear to be more easily accepted than lines containing numerous genes (e.g., *OsYSL2*–*HvNAS1*–*ferritin*; Masuda et al., 2012).

A feeding test of biofortified rice in mice and Caco-2 (human epithelial colorectal adenocarcinoma cells) cell lines confirmed that the increased metal content of rice owing to increased NAS expression is highly bioavailable (Lee et al., 2009b, 2011, 2012b; Zheng et al., 2010). These results suggest that at least in the case of NAS overexpression lines, there is no need to co-express phytase or metalloproteins to increase bioavailability. Recently it was shown that biofortification of rice with Zn significantly increases Zn uptake in Caco-2 as well as in rat pups, and are suggested to be the same in human populations (Jou et al., 2012). Feeding tests in humans may be the next step toward the release of biofortified lines. In addition to the genes and transgenic lines already available in different breeding programs, new genes and new lines with better combinations are being sought. Micronutrient-biofortified rice may be bred with golden rice and/or folate-rich rice to provide even more essential nutrients and vitamins.

## Conflict of Interest Statement

The authors declare that the research was conducted in the absence of any commercial or financial relationships that could be construed as a potential conflict of interest.
